# Porcine and Canine von Willebrand Factor and von Willebrand Disease: Hemostasis, Thrombosis, and Atherosclerosis Studies

**DOI:** 10.1155/2010/461238

**Published:** 2011-02-07

**Authors:** Timothy C. Nichols, Dwight A. Bellinger, Elizabeth P. Merricks, Robin A. Raymer, Mark T. Kloos, Natalie DeFriess, Margaret V. Ragni, Thomas R. Griggs

**Affiliations:** ^1^Department of Medicine, Francis Owen Blood Research Laboratory, University of North Carolina, Chapel Hill, NC 27516, USA; ^2^Pathology and Laboratory Medicine, Francis Owen Blood Research Laboratory, University of North Carolina, Chapel Hill, NC 27516, USA; ^3^Division of Laboratory Animal Medicine, Francis Owen Blood Research Laboratory, University of North Carolina, Chapel Hill, NC 27516, USA; ^4^Medicine/Hematology/Oncology, University of Pittsburgh Medical Center, Pittsburgh, PA 15261, USA; ^5^Hemophilia Center of Western PA, Pittsburgh, PA 15213, USA

## Abstract

Use of animal models of inherited and induced von Willebrand factor (VWF) deficiency continues to advance the knowledge of VWF-related diseases: von Willebrand disease (VWD), thrombotic thrombocytopenic purpura (TTP), and coronary artery thrombosis. First, in humans, pigs, and dogs, VWF is essential for normal hemostasis; without VWF bleeding events are severe and can be fatal. Second, the ADAMTS13 cleavage site is preserved in all three species suggesting all use this mechanism for normal VWF multimer processing and that all are susceptible to TTP when ADAMTS13 function is reduced. Third, while the role of VWF in atherogenesis is debated, arterial thrombosis complicating atherosclerosis appears to be VWF-dependent. The differences in the VWF gene and protein between humans, pigs, and dogs are relatively few but important to consider in the design of VWF-focused experiments. These homologies and differences are reviewed in detail and their implications for research projects are discussed. The current status of porcine and canine VWD are also reviewed as well as their potential role in future studies of VWF-related disorders of hemostasis and thrombosis.

## 1. Introduction

Animal models of von Willebrand factor (VWF) deficiency, both inherited and induced, have provided key insights into VWF-related diseases. This paper focuses on the current status of knowledge of porcine and canine VWF and von Willebrand disease (VWD) and how investigations with these animals have advanced the understanding of the seemingly paradoxical roles of VWF in hemostasis and thrombosis as well as atherogenesis [1]. 

**Figure 1:**
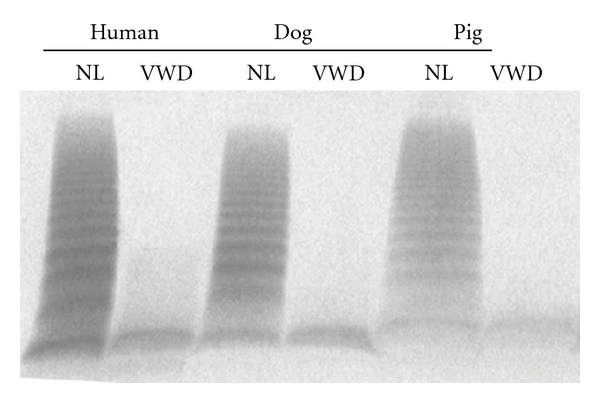
Multimer distribution of von Willebrand factor antigen in normal (NL) and von Willebrand disease (VWD) human, dog, and pig plasma samples. Human plasmas were obtained from George King Bio-Medical, Inc., Overland Park, Kansas. The human VWD plasma was from a patient with severe type 3 VWD plasma with VWF : RCo 15 IU/dl and VWF : Ag 1 IU/dl. The dog and pig plasmas were prepared at the Francis Owen Blood Research Laboratory at the University of North Carolina at Chapel Hill using normal and severe VWD animals that had no detectible activity or antigen in either species. None of the subjects had recently been transfused with VWF-containing products. Anti-VWF antibodies for immunostaining were purchased from Dako (A082, Carpintera, CA) (1.5% agarose gel) [7–9]. The identity of the very bottom band seen in all lanes is unknown but is a consistent finding in multiple laboratories and is also seen in murine VWD plasma [7–11]. It is possible that this band simply represents nonspecific binding of the antibody to the leading edge of the proteins at the end of the electrophoresis procedure.

In humans, pigs, and dogs, VWF is a large, adhesive glycoprotein that supports hemostasis by mediating platelet adhesion to injured vessel surfaces. Porcine and canine VWD mirror type 3 human VWD, phenotypically exhibiting a severe mucosal and cutaneous bleeding diathesis that most often involves nasal, oral, gastrointestinal, and genitourinary mucosa. Without prompt treatment, bleeding can be crippling or fatal in all three species. In addition, humans and animals with VWD are often recognized when excessive bleeding occurs in association with relatively minor trauma or surgery. Affected pigs and dogs have undetectable VWF antigen, activity, and multimers (Figure 1, Table 1). The inheritance pattern across species occurs in an autosomal fashion [2–6]. Understanding the molecular and biochemical similarities and differences between human, porcine, and canine VWF and VWD has proven seminal in characterizing critical protein-protein interactions involved in VWF-mediated hemostasis and thrombosis.

## 2. Porcine VWF and VWD

The porcine VWF gene is on pig chromosome 5q21, a region that is syntenic with human chromosome 12p13.2, the location of the human gene [12, 13]. The human VWF gene is located near the telomere, perhaps in part accounting for the high frequency of mutations resulting in various types of VWD, while the pig VWF gene is more centromeric. Porcine VWD appears to be among the oldest recognized animal models of an inherited bleeding disorder [14]. The molecular defect in porcine VWD has been linked to the VWF locus, but the precise molecular mechanism has remained elusive [15].

**Figure 2:**
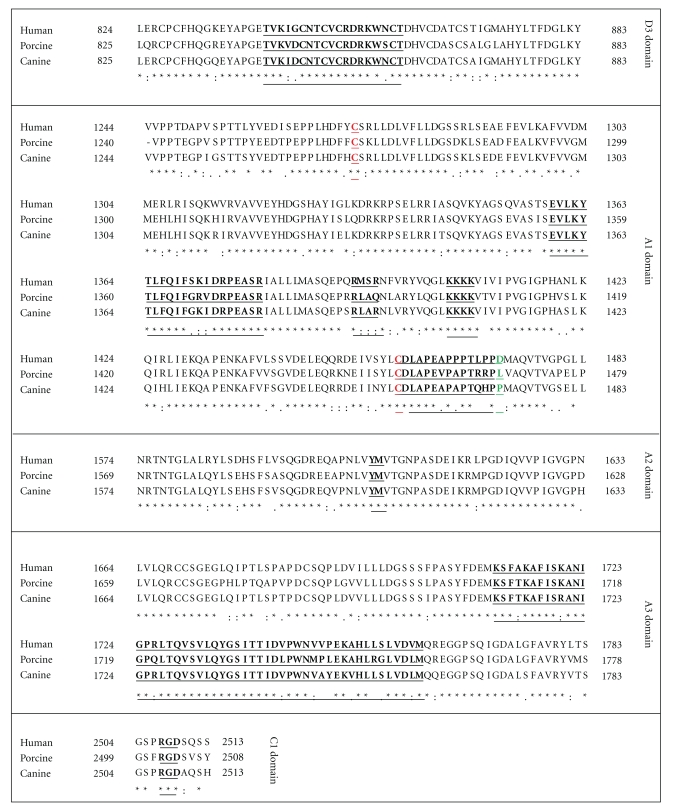
Alignments of Human, Porcine, and Canine VWF. (a) Region of D′/D3 domain highlighting the F.VIII : C binding region (underlined). (b) Region of A1 domain showing the conserved 1272–1458 disulfide bonds (C1272 and C1458) in red and GP1b binding sites in black underlined. The D1472H human polymorphism site is highlighted in green. (c) Region of A2 domain showing the ADAMTS13 cleavage site (underlined). (d) Region of A3 domain highlighting Collagen binding site. (e) Region in C1 domain indicating the RGD binding site of integrin *α*IIb/*β*III. The VWF amino acid sequences were analyzed by Clustal W multiple sequence alignment program [16] and derived from NCBI Accession NP_000543 (Human), AF052036 and AY004876 (Porcine), and NP_001002932 (Canine).

The complete cDNA sequence of porcine VWF predicts a protein of 2,807 amino acids, six residues shorter than human VWF (Figure 2) [17–19]. Porcine and human VWF share 84.3% identity, with additional 7% conservative substitutions. The propeptide and mature portions of porcine VWF are equally homologous to the human sequence. Both human and porcine pre-pro-VWF contain 234 cysteines conserved in perfect register. Vicinal cysteine motifs (CXXC) thought to be involved in disulfide isomerase activity [20] are also conserved in D1, D2, and D3 domains of each of these species. Using the human numbering system for reference, the following residues are deleted from porcine VWF: G215 (mid-D1 domain), GGLV1241–1244 (D3/A1 domain boundary), and 1494S (A2 domain). The deleted residues 1241–1244 are located within a negatively charged region flanking the amino terminal side of the A1 domain disulfide loop that is involved in interaction with the platelet-binding agonist, ristocetin. This region is also critical to the regulation of VWF binding to platelet GP1b under physiologic conditions and maintenance of the A1 loop in an unreactive configuration. Porcine plasma VWF spontaneously binds human platelets in the absence of an agonist such as ristocetin or botrocetin, and the deletion of these four residues in this regulatory area of human VWF-GP1b binding may help explain this observation [2, 4, 17, 21–23]. Interestingly, botrocetin supports pig VWF and pig platelet agglutination but ristocetin does not [4, 21–23]. Botrocetin also supports agglutination of pig platelets by human VWF [22]. In humans, a D1472H polymorphism is associated with decreased ristocetin activity, and pigs have a leucine at the homologous position (Figure 2) [24]. The pig platelet Gp1b sequence is conserved (not shown) suggesting the D1472L substitution in pig VWF may, in part, account for the relative lack of ristocetin reactivity. The RGD sequence is conserved and likely mediates platelet integrin *α*IIb*β*3 binding (Figure 2). Recombinant porcine VWF trafficked to storage granules, is efficiently multimerized and secreted, and spontaneously agglutinates human platelets [18].

## 3. Canine VWF and VWD

The canine VWF gene has been localized to canine chromosome27 (http://www.ncbi.nlm.nih.gov/gene/399544) in a region that is syntenic with human chromosome 12p13.2. Like human VWD, canine VWD appears to be the most prevalent inherited bleeding disorder in various strains of dogs, and, thus, there is significant interest in detection and characterization of the disease and finding new treatments in all species [3, 31–34]. The Chapel Hill, canine VWD strain was derived from a show-dog strain of Scottish Terriers and this colony was established in 1978 from a single heterozygote [4]. This causative mutation in the Chapel Hill strain of canine type 3 VWD is due to a single nucleotide deletion in the canine VWD sequence at base pair 255 that causes a frameshift and a premature stop codon in exon 4 [35]. These VWD dogs make no detectable VWF mRNA or protein.

The complete cDNA sequence of canine VWF predicts a protein of 2,813 amino acids and thus is identical in the number of amino acids to human VWF (Figure 2) [36, 37]. The full-length human and canine VWF are 87.1% identical at the nucleotide level and 86.2% at the protein level, with an additional 6% conservative substitutions. The propeptide and mature portions of canine VWF are 87.3 and 92.8% homologous, respectively, to the human sequence. Like porcine VWF, canine pre-pro-VWF contains 234 cysteines conserved in perfect register with the homologous portion of human VWF. Vicinal cysteine motifs (CXXC) are also conserved in D1, D2, and D3 domains of each of these species. Canine VWF does not react with ristocetin; botrocetin however, is a reliable canine VWF activator and supports the agglutination of canine platelets by canine and human VWF [4, 21, 22]. The reason for the lack of ristocetin reactivity may in part be explained by the D1472P amino acid substitution in canine VWF similar to that in porcine VWF as described above (Figure 2) [24]. The dog platelet Gp1b sequence has an M239T substitution that does not appear to have a “gain of function” substitution as reported for the M239V mutation in human platelet GP1b [24]. It is also possible that botrocetin more specifically activates VWF than does ristocetin [38]. The RGD sequence is conserved and most likely mediates platelet integrin *α*IIb*β*3 binding in humans, pigs, and dogs. Recombinant canine VWF established VWF-dependent Weibel-Palade body formation in cultured primary VWD endothelial cells [35].

## 4. Research Utilizing VWD Pigs and Dogs

### 4.1. Replacement Products

Pigs and dogs with VWD have been used to determine safety, efficacy, pharmacodynamics, and pharmacokinetics in the preclinical testing of plasma-derived and recombinant human VWF (rhVWF) [10, 39–42]. Importantly, rhVWF reduces the saline bleeding time [25] from >15 minutes to ~5 minutes [10]. The correction of the saline bleeding time may portend support of hemostasis when rhVWF is given to VWF-deficient humans.

Infusion of plasma-derived and recombinant human VWF into VWD pigs and rhVWF into VWD dogs is accompanied by a delayed rise in factor VIII activity (F.VIII). Conversely, when human F.VIII is infused into VWD dogs, the half-life of the infused F.VIII is markedly reduced when compared to infusing the same amount of human F.VIII into hemophilia A dogs with normal VWF levels [43]. Taken together, these data are consistent with human VWF binding to and stabilization of F.VIII in both species *in vivo*.

In VWD dogs, the half-life of rhVWF is between 10.2 and 13.0 hours [10] and the half-life of plasma-derived canine VWF is between 12 and 18 hours [44]. In VWD pigs, the half-life of rhVWF is ~10 to 16 hours [11, 39], and the half life of plasma derived porcine VWF is between 10 and 18 hours [11, 45]. Thus, both pigs and dogs with VWD appear to clear human and species-specific VWF in a comparable fashion. The results of preclinical testing of rhVWF have been predictive of the half-life found in early testing in human subjects [46].

### 4.2. VWF Secretion with DDAVP and VWF mRNA Upregulation by rhIL-11

Recombinant human IL-11 (rhIL-11, Neumega), a glycoprotein 130- (gp130-) signaling cytokine that is approved for treatment of thrombocytopenia, has been shown to induce elevations in VWF and F.VIII in humans and mice [47]. In these initial studies, it was unclear if the mechanism for VWF elevation was mediated by increased secretion or increased production by upregulation of VWF mRNA. If the latter mechanisms were shown to be correct, then rhIL-11 could be used as an additional or alternative therapy to 1-desamino-8-D-arginine vasopressin (DDAVP), a standard therapy for increasing VWF levels in humans through the Weibel-Palade body secretory pathway [34, 48, 49]. To compare DDAVP and rhIL-11, dog models were informative since canine but not murine species respond to DDAVP. rhIL-11 produces a gradual, sustained elevation of VWF and F.VIII levels in both normal and heterozygous VWD (VWF +/−) dogs, while DDAVP produces a rapid, nonsustained increase. rhIL-11 treatment produces a 2.5 to 11-fold increase in VWF mRNA in normal dogs but not in homozygous (VWF −/−) VWD dogs, thus identifying a mechanism for elevation of plasma VWF *in vivo*. Moreover, dogs pretreated with rhIL-11 retain a DDAVP-releasable pool of VWF and F.VIII, suggesting that rhIL-11 does not significantly alter trafficking of these proteins to or from storage pools. The half-life of infused VWF is unchanged by rhIL-11. These results strongly suggest that rhIL-11 and DDAVP raise plasma VWF levels by different mechanisms, and that rhIL-11, like DDAVP, could be an alternative to plasma-derived products for some VWD or hemophilia A patients who are unresponsive to DDAVP or in whom DDAVP is contraindicated [50]. Likewise, rhIL-11 therapy could also reduce use of plasma products in some patients. It is important to bear in mind that DDAVP causes an immediate release of VWF, and tachyphylaxis limits repeated dosing in the short term. In contrast, rhIL-11 induces a sustained increase of VWF that, once established over a few days, appears to persist until administration is discontinued. Thus, the two drugs may well be complementary in many patients if shown to be safe in clinical trials.

Recently, rhIL-11 was administered subcutaneously to nine subjects with mild or type 1 VWD [51] (5F, 4M, age 21–49 yr) in an FDA-approved phase 2 open label, dose escalation study [7]. The drug was well tolerated and these data confirm that rhIL-11 increases VWF in humans with mild or type 1 VWD by means other than the DDAVP releasable pool of VWF, and possibly by increasing VWF mRNA. To our knowledge, if this mechanism was confirmed in additional studies, rhIL-11 would be the first medication for treating an inherited bleeding disorder that actually targets the relevant gene *in situ*. These encouraging results have provided the foundation for obtaining FDA permission to perform two phase 2 efficacy studies in mild or type 1 VWD patients, the first in patients with menorrhagia and the second in patients undergoing elective surgery. These ongoing studies will determine if the results with rhIL-11 therapy found in the VWD dogs and the phase 2 dose escalation study can be translated into a safe, new treatment for VWF-deficient humans.

### 4.3. Atherosclerosis

The role of VWF in atherosclerosis has been a subject of debate. A mechanism linking VWF to atherosclerosis is based on the fact that VWF mediates platelet adhesion to injured arterial walls, thus delivering platelet contents, including platelet-derived growth factor (PDGF), to the arterial wall. The hypothesis is that relatively high concentrations of PDGF promote atherogenesis at focal points of vascular injury [52–54]. The corollary is that the absence of VWF reduces atherogenesis. 

Since dogs tend not to get atherosclerosis, pigs are a more appropriate animal model in which to test these hypotheses [55]. To date, variations in the degree of coronary and abdominal aortic atherosclerosis have been reported for normal and VWD pigs [56–64]. However, these results seem to be impacted by cholesterol levels. In a retrospective study, polymorphisms in the apolipoprotein B100 genotype were found to significantly influence the severity of high-fat diet-induced atherosclerotic plaque formation in VWD and normal swine without regards to the VWD genotype [65]. Humans with various types of VWD including type 3 are not protected from developing coronary and aortic atherosclerosis but may develop fewer occlusive thrombi with ensuing organ infarction [66, 67]. Thus, VWF may mediate thrombotic complications of atherosclerosis rather than atherogenesis *per se* as discussed next.

### 4.4. VWD and Occlusive Arterial Thrombosis

The potential role of VWF in the development of arterial thrombosis noted in VWD humans with atherosclerosis has been studied in pigs and dogs. VWD pigs with or without atherosclerosis and VWD dogs do not develop occlusive arterial thrombosis in the Folts stenosis and injury model [8, 64, 68]. In addition, neutralizing VWF activity interrupts VWF-dependent arterial thrombosis in nonatherosclerotic pigs with normal VWF expression [69, 70]. Several molecules that inhibit VWF activity have been developed as potential therapeutic agents, and this work has recently been reviewed [71]. One of the most promising novel approaches is the aptamer ARC1779 [72–74]. Aptamers are synthetic nucleotides that bind with high affinity to a target protein and neutralize its function. The current status of the VWF-binding aptamer ARC1779 suggests that it will be a safe and effective new therapeutic that addresses unmet needs for treating patients with arterial thrombotic diseases, particularly those that are VWF mediated [75].

VWF serves as a carrier for plasma F.VIII in humans, pigs, and dogs and thereby protects F.VIII from degradation by activated protein C [76]. This carrier function may also deliver F.VIII to sites of arterial injury and localize F.VIII activity to sites of VWF and platelet attachment on exposed subendothelium and promote thrombosis [77]. In addition, hemophilia A dogs (i.e., no detectable F.VIII but normal VWF) form occlusive arterial thrombosis as readily as normal dogs using the same experimental model [8]. In pigs, neutralizing VWF activity while leaving F.VIII activity intact prevents the development of occlusive thrombosis [69]. Human subjects with hemophilia A that lack F.VIII have serious impairment of thrombin generation but are not protected from myocardial infarction or thrombosis complicating aortic atherosclerosis [78–80]. Moreover, VWF level in humans also correlates directly with thrombosis risk and inversely with bleeding risk [1]. These results support the hypothesis that VWF has an intrinsic property that supports arterial thrombosis independent of its association with F.VIII in humans, pigs, and dogs.

### 4.5. VWD and Bleeding Time Prolongation

In humans, pigs, and dogs, VWF is synthesized in endothelial cells that store and secrete the protein into the subendothelium and plasma (Table 1) [81, 82]. VWF is also synthesized in human and porcine megakaryocytes and is present in human and porcine platelet alpha granules [27, 83–85]. VWF does not appear to be synthesized in canine megakaryocytes nor is it present in platelet alpha granules [8, 35]. Studies in the three species have shown that bleeding time prolongation is variably affected by decreased platelet VWF and plasma VWF [9, 27, 86, 87]. In porcine VWD and human type 3 VWD, replacement of both plasma and platelet VWF by transfusion is required to normalize the bleeding time, whereas plasma VWF alone is insufficient [9, 27, 88–90]. Dogs that have no platelet VWF but normal plasma and endothelial VWF have normal bleeding times [8]. In contrast to humans and pigs with VWD, infusion of canine VWF into VWD dogs nearly normalizes bleeding time [8]. Thus, bleeding time, as a measure of hemostasis, appears to have species-specificity related to VWF distribution in plasma, platelets, and endothelium.

 Organ transplantation can transfer the entire VWF gene *in situ* and provides a model to study VWF function in various compartments (Table 1). For example, bone marrow transplantation in porcine VWD allows for separate expression of VWF in the platelet or plasma, endothelial, and subendothelial compartments [9, 27, 28]. In these studies, plasma VWF alone was sufficient to support bleeding time and the development of occlusive arterial thrombosis in the Folts arterial stenosis and injury model [9]. Likewise, transplantation of normal liver and lungs into VWD pigs has provided circulating VWF without platelet VWF [29, 91]. The transplanted normal liver provides sufficient amounts of plasma VWF to correct the bleeding time from >15 minutes to ≤5 minutes [26]. The transplanted normal lung, however, provides ~5% of normal VWF but is insufficient to correct the prolonged bleeding time. Thrombosis studies have not been performed in the liver and lung transplantation experiments. Thus, bleeding time prolongation in pigs appears to occur when VWF is markedly decreased or absent from the plasma and/or subendothelial compartments. The precise role of platelet VWF in this scenario is unknown.

### 4.6. Gene Transfer

An inherited bleeding disorder such as VWD is an attractive target for gene transfer using viral vectors for several reasons. First, there is a single gene defect. Second, the protein can be expressed from many target organs that will secrete the transgene product into plasma. Third, successful gene transfer would reduce the requirement for blood products and the associated invasive procedures required for administration which could very realistically be expected to improve patient comfort and well-being. Fourth, expression of VWF at relatively low levels may provide some degree of phenotypic correction from bleeding; overexpression, however, potentially could be associated with thrombotic side effects [1]. Contemporary challenges for gene therapy are well recognized and VWF is no exception [92–94]. Nonetheless, human VWF has been expressed in blood outgrowth endothelial cells isolated from VWD dogs [95], and progress has been made with murine models [96, 97]. The availability of porcine and canine VWF cDNA and newer gene transfer vectors provides an opportunity to transfer this work to large animal models that have a recognized strong preclinical predictive value [98].

### 4.7. Lung Xenotransplantation and VWD

Separate from the role of VWF in hemostasis and thrombosis, lung transplantation studies have focused on the binding of porcine VWF and primate xenoreactive antibodies. Swine pulmonary xenografts, as opposed to cardiac and renal xenografts, release large amounts of porcine VWF when transplanted into nonhuman primates. Human and nonhuman primates have xenoreactive antibodies that bind to carbohydrate side chains on porcine VWF [99]. Also, porcine VWF spontaneously agglutinates human platelets [2]. The absence of porcine VWF in VWD donor lungs results in longer survival of the transplant in nonhuman primates possibly due to mechanisms mediated by reduced or absent VWF-mediated xenoantibody binding and platelet agglutination [100]. These findings strongly support the hypothesis that porcine VWF is a mediator of pulmonary xenograft dysfunction and is a potential drug target for improving xenograft survival.

### 4.8. Thrombotic Thrombocytopenia Purpura (TTP)

Discovery of the roles of VWF and its cleavage enzyme ADAMTS13 in TTP has revolutionized the understanding of the underlying mechanisms that mediate this previously poorly understood disorder [101]. In studies performed prior to this discovery, normal pigs and dogs injected with botrocetin developed thrombocytopenia, microthrombi in lungs and spleen but not kidney or brain, and initial depletion of VWF multimers followed by the appearance of ultra large VWF multimers during recovery. VWD pigs and dogs were not affected by botrocetin infusion suggesting that plasma VWF mediates this botrocetin-induced thrombotic thrombocytopenia in the absence of platelet VWF [102]. Since ADAMTS13 had not been discovered when these studies were performed, it was not assayed in these animals. Recently, infusion of shiga toxin has been shown to support the development of a thrombotic microangiopathy in ADAMTS13-deficient mice, but the effect of the infusing botrocetin on ADAMTS13 or in the absence of ADAMTS13, if any, is unknown [103–106]. The ADAMTS13 cleavage site is preserved in both porcine and canine VWF (Figure 2). It is possible, then, that expression of porcine or canine VWF in VWD pigs or dogs with mutations in the ADAMTS13 cleavage site, respectively, could result in a TTP syndrome. Application of such information to the human condition would need to consider the possibility that characterization of ADAMTS13 is incomplete in many species and that important functional differences may exist between them [107].

 Importantly, a novel approach to the treatment of TTP in humans has been to neutralize VWF activity with the aptamer ARC1779 [108, 109]. Administration of this aptamer was well tolerated and appeared to be associated with a significant increase in platelet counts in a case of refractory TTP. Currently, ARC1779 is being tested as an “add-on” therapy to plasma exchange in patients with TTP.

### 4.9. Endocarditis Susceptibility

VWD swine appear to be resistant to experimentally induced group C streptococcal endocarditis, whereas normal pigs readily develop the infection [110]. These data suggest that normal VWF-mediated platelet function is necessary for establishing this particular infection. Understanding of platelet function in inflammation and infection is evolving rapidly, and pigs and dogs with VWD likely will have a role in these studies [111–113].

## 5. Summary and Conclusions

Pigs and dogs with VWD have provided faithful phenotypic models for the study of basic aspects of human type 3 VWD, as well as powerful tools for revealing pathogenesis, developing new therapies, and testing their safety and efficacy. This conclusion is remarkable, considering the important differences in porcine and canine VWF amino acid sequence and reactivity to agonists such as ristocetin and botrocetin. Both pigs and dogs, however, have proven to be attractive species for studying VWF structure-function relationships and VWF interactions with F.VIII, platelet GP1b and *α*IIb*β*3, collagen, and shear stress. In addition, these animals have provided key insights into the roles of platelet and plasma VWF in hemostasis and thrombosis. The availability of full-length porcine and canine VWF cDNAs opens the possibility of expressing VWF with disease-causing mutations that recapitulate other types of VWD, as recently initiated in mice [114, 115]. Likewise, the recent cloning of murine VWF cDNA and correction of bleeding time prolongation in VWD mice by murine VWF will also provide important mechanistic insights into the primary role of VWF in hemostasis [116, 117]. The extensive characterization of VWF and VWD in pigs, dogs, and mice will allow investigators to choose the most appropriate model to answer the question at hand. Improved understanding of VWF-mediated hemostasis and thrombosis in all such animals will continue to contribute to better modeling and understanding of human bleeding, thrombotic, and cardiovascular diseases.

## Figures and Tables

**Table 1:** Differential correction of phenotype of VWD swine by transplantation of liver, kidney, bone marrow, and lung.

		VWF					

Transplant procedure	Cell(s) synthesizing VWF	Plasma	Platelet	F.VIII	Bleeding time [25]	Thrombosis	References

None	None	None	None	Low	Prolonged	Not done	[14, 25]
Normal liver to VWD	Hepatic endothelium	Near normal to normal	Not done	Near normal to normal	Near normal to normal	Not done	[26]
Normal kidney to VWD	Presumed endothelium	None	Not done	Low	Prolonged	Not done	[26]
Normal bone marrow to VWD	Megakaryocyte	Little to none	Present	Low	Prolonged	No	[9, 27, 28]
VWD bone marrow to normal	Endothelium	Normal	Absent	Normal	Near normal to normal	Yes	[9, 28]
Normal Lung to VWD	Pulmonary endothelium	~5%	Absent		Prolonged	Not done	[29]

Adapted with permission from Brinkhous KM, Reddick RL, Read MS, Nichols TC, Bellinger DA, Griggs TR. von Willebrand factor, and animal models: contributions to gene therapy, thrombotic thrombocytopenic purpura, and coronary artery thrombosis. Mayo Clin Proc. 1991; 66 : 733–742 [30].
